# Rare case of longevity in Hutchinson-Gilford progeria syndrome and literature review

**DOI:** 10.1186/s13023-025-04022-6

**Published:** 2025-10-07

**Authors:** Xiao-ling Cai, Hang Chen, Xue-qin Lin, Hong Zhang, Yi-fei Xiang, Ming-xiang Wu, Kai-yang Lin

**Affiliations:** 1 Department of Cardiology, Shengli Clinical Medical College of Fujian Medical University, Fujian Provincial Hospital, Fuzhou University Affiliated Provincial Hospital, Fuzhou, China; 2 Department of Cardiology, Xiapu County Hospital, Ningde, Fujian Province China

**Keywords:** HGPS, LMNA gene, NSTEMI, Heart failure

## Abstract

Hutchinson-Gilford progeria syndrome (HGPS) is a rare autosomal dominant disorder characterised by premature ageing, with an average life expectancy of 14.6 years. We report a case of HGPS associated with a typical C. 1824 C > T (P. Gly608Gly) mutation in the 11th exon of the LMNA gene in a 21-year-old woman. The patient presented with a three-year history of progressive exertional dyspnea that acutely worsened over the five days preceding admission. She had a short stature (weight 13 kg, height 85 cm), typical craniofacial features, and scleroderma-like skin changes. Cardiovascular evaluation showed signs of premature ageing (ejection fraction 30.8%). This patient is the oldest among all reported cases of HGPS associated with typical mutations. We describe rapid progression of HGPS in this patient and recommend that physicians should consider coronary heart disease in the differential diagnosis of chest pain in patients with HGPS, regardless of age.

**Supplementary Information:**

The online version contains supplementary material available at 10.1186/s13023-025-04022-6.

## Introduction

Based on previous research, the incidence of Hutchinson-Gilford progeria syndrome (HGPS), a rare sporadic autosomal dominant genetic disorder [1], is roughly 1 in 4–8 million live births [2]. The average life expectancy of children with HGPS is approximately 14.6 years [3]. Multiple organs may be affected by HGPS, and cutaneous signs are frequently the first symptom that patients develop [4]. HGPS is diagnosed based on clinical features and genetic analysis, which confirms *LMNA* gene mutations [5]. We report a case of HGPS associated with a typical mutation in the 11th exon of the *LMNA* gene in a 21-year-old woman.

## Case report

**Fig. 1:**
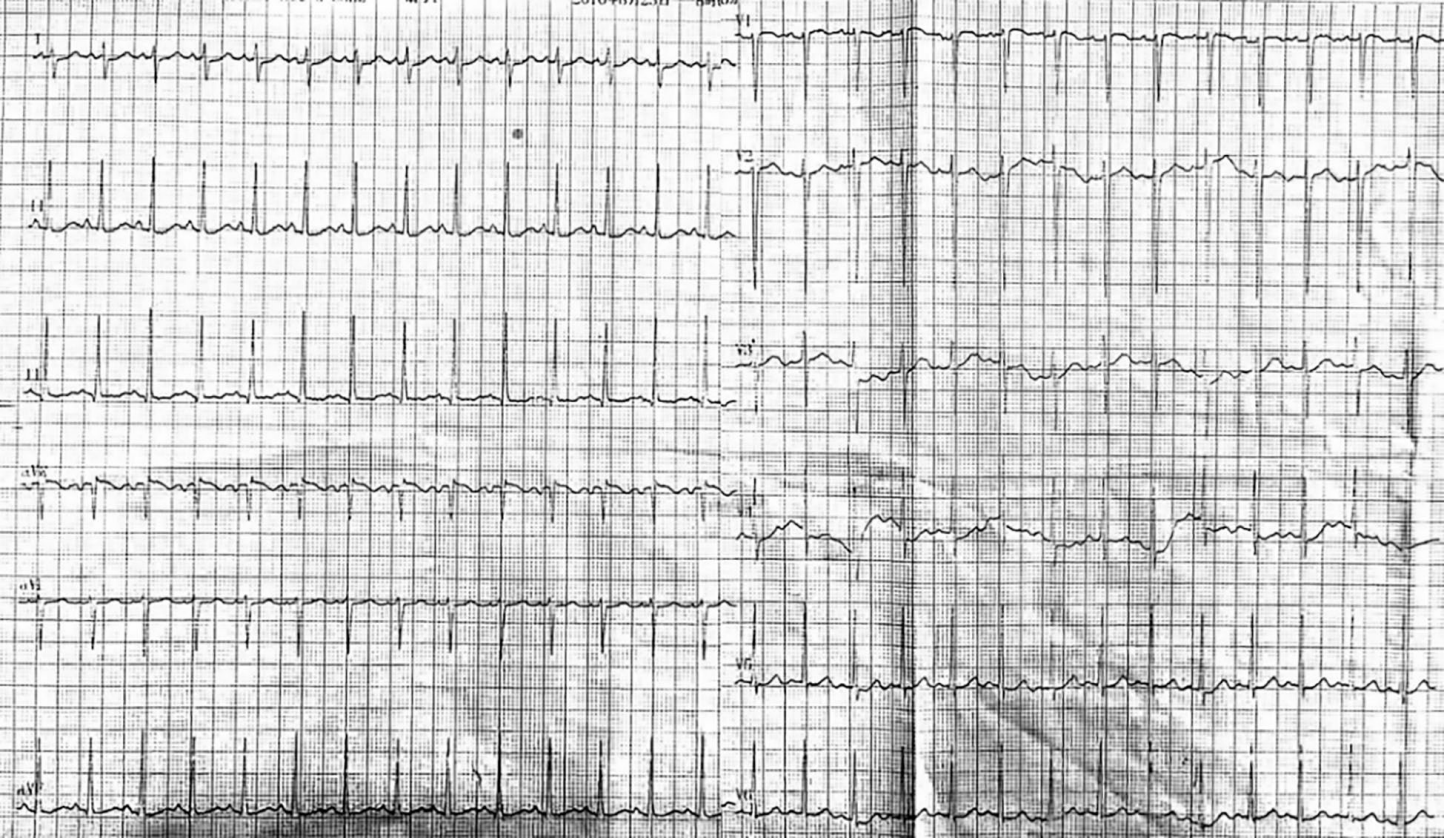
Electrocardiography revealed sinus tachycardia and no ST-T segment elevation

A 17-year-old adolescent female was admitted to our hospital for evaluation of a greater than 2-week history of dull episodic chest pain that radiated to the shoulder and back. She had 2–3 episodes of pain accompanied by palpitations and shortness of breath each day, and each episode lasted approximately 5 min. Her mother reported that the patient was born prematurely at 7 months of gestation. She had skin edema at 3 years of age and was diagnosed with “scleroderma.” She gained height and weight at a slow rate from an early age. She had an upper respiratory tract infection 1 month prior to admission. On physical examination, she was 85 cm tall and weighed 12 kg. She showed characteristic facial features including a disproportionate head-to-face ratio, scalp vein protrusion, falling out of hair, eyebrows, and eyelashes, protruding eyes, a narrow nasal bridge, thin lips, and mandibular retrusion. We observed abdominal protrusion with pigmentation and scleroderma-like skin changes. Laboratory test results showed an increased white blood cell count (13.8 × 10^9^/L; reference range, 3.5–9.5/L), neutrophilic granulocyte percentage (76.9%; reference range, 40–75%), platelet count (539 × 10^9^/L; reference range, 125–350/L), and serum N-terminal pro-B-type natriuretic peptide (NT-proBNP) (254 ng/mL; reference range, 0-300 ng/mL) and troponin I (0.369 ng/ml; reference range, 0-0.04 ng/ml) levels. Electrocardiography revealed sinus tachycardia and no ST-T segment elevation (Fig. 1). Echocardiography revealed decreased left ventricular diastolic function and aortic valve and bilateral coronary artery calcification. Based on the patient’s history of upper respiratory tract infection before admission and her young age, we considered a diagnosis of viral myocarditis.

**Fig. 2:**
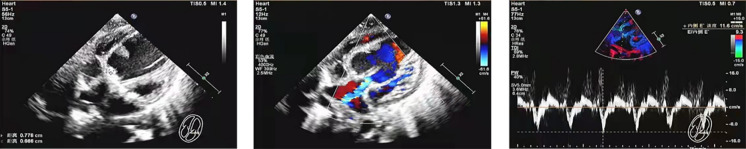
Echocardiography revealed left atrial and ventricular enlargement (left atrial diameter 21.7 mm, and left ventricular diameter 48.7 mm), left ventricular segmental wall motion abnormalities, left ventricular systolic insufficiency (EF:30.8%) and diastolic dysfunction, moderate mitral regurgitation, and hyperechoic nodules (the largest approximately 7.8 mm×6.7 mm in size) in the interventricular septum and left ventricular wall

**Fig. 3:**
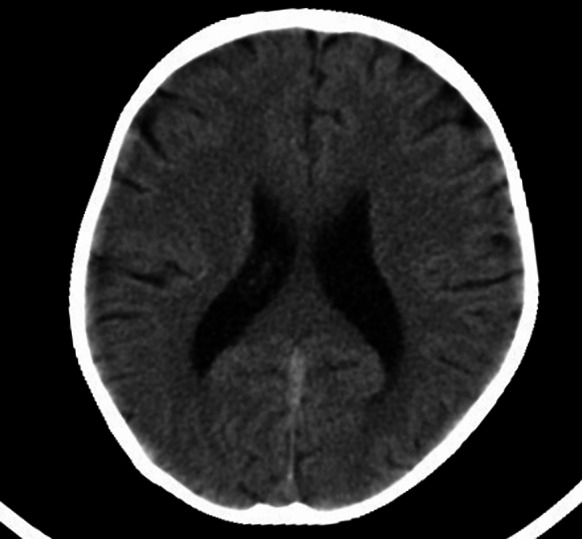
Computed tomography examination showed bilateral ventricle enlargement and sulci widening

The patient was readmitted in October 2021 for evaluation of a 3-year history of shortness of breath after activity, which had worsened 5 days prior to presentation, accompanied by abdominal distension and oedema of bilateral lower extremities. Although she was 21 years old, she weighed only 13 kg and was 85 cm tall. At the time of the current admission, the patient’s skin appeared significantly worse than on her first admission, hair loss was more pronounced, and her scalp veins and eyes were more prominent secondary to severe subcutaneous fat atrophy. Cardiac auscultation revealed that the second heart sound heard over the pulmonary valve area was greater than that over the aortic valve region, and a 3/6 systolic murmur was auscultated over the areas corresponding to the mitral and tricuspid valves. Blood test results revealed an increase in the white blood cell count (16.2 × 10^9^/L), neutrophilic granulocyte percentage (83.3%), and platelet count (487 × 10^9^/L). The serum troponin I level was 2.101 ng/mL, and the serum NT-proBNP measured 23,100 ng/L, which suggested severely impaired cardiac function. Echocardiography revealed left atrial and ventricular enlargement (left atrial diameter 21.7 mm, and left ventricular diameter 48.7 mm), left ventricular segmental wall motion abnormalities, left ventricular systolic insufficiency (EF: 30.8%) and diastolic dysfunction, moderate mitral regurgitation, and hyperechoic nodules (the largest approximately 7.8 mm×6.7 mm in size) in the interventricular septum and left ventricular wall (Fig. 2). Computed tomography revealed encephalatrophy (Fig. 3) and atherosclerosis involving several blood vessels throughout the body.

**Fig. 4:**
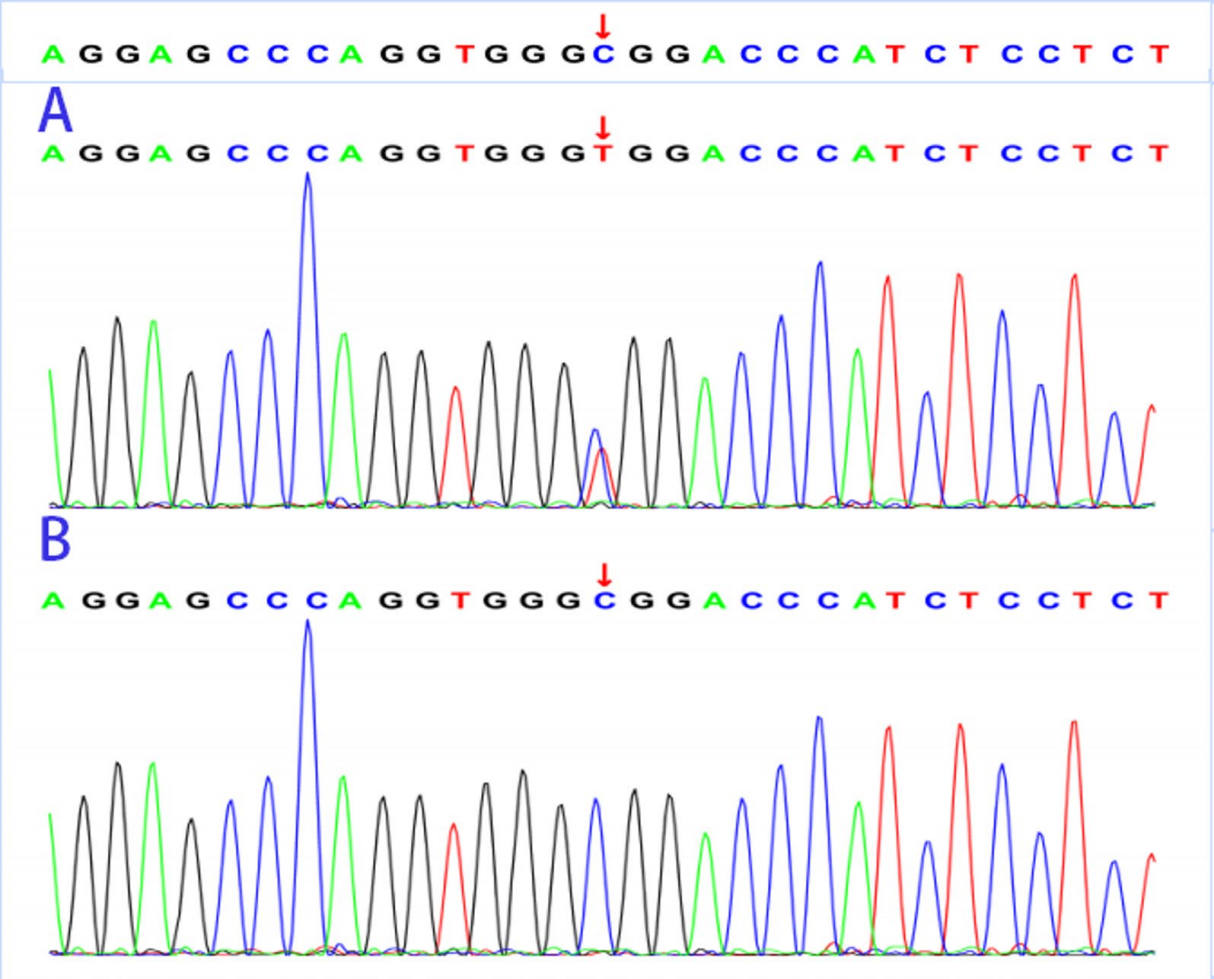
LMNA gene sequencing results of the patient and her families. The patient had a typical C. 1824C>T (P. Gly608Gly) mutation in the 11th exon of the LMNA gene (**A**), which was not found in the rest of the family (**B**)

Based on these findings, the patient was diagnosed with acute decompensated heart failure, and we initiated symptomatic treatment including cardiotonic and diuretic administration. Considering the facial features and evidence of premature aging of the cardiovascular and nervous systems, we suspected progeria in this case and obtained blood samples from the patient and her family members for genetic testing, following informed consent from all individuals involved in the study. Gene analysis showed a typical C. 1824 C > T (P. Gly608Gly) mutation in the 11th exon of the LMNA gene only in the patient (Fig. 4). The patient’s genetic analysis results and typical facial features confirmed the diagnosis of HGPS. We reviewed the patient’s history of chest pain 5 years prior and compared the echocardiographic images obtained at that time to conclude that her previous chest pain was possibly attributable to non-ST-elevation myocardial infarction (NSTEMI). A literature review revealed that currently lonafarnib is the mainstay of treatment for HGPS; therefore, we have actively applied for Lonafarnib treatment for the patient in an attempt to prolong survival.

## Discussion

We report the case of a 21-year-old Chinese adolescent female with HGPS, chronic heart failure, and NSTEMI, who presented with typical craniofacial features and evidence of premature ageing of the cardiovascular system. Genetic testing confirmed a typical C. 1824 C > T (P. Gly608Gly) mutation in the 11th exon of LMNA gene. Subsequently, we searched PubMed, Web of Science, Embase, and Cochrane databases and found that our patient was the oldest currently reported HGPS patient with a typical mutation of C. 1824 C > T (P. Gly608Gly) (Table 1, Supplementary Table 1).

**Table 1:** Literature review of Hutchinson-Gilford Progeria syndrome

cases		Case 1	Case 2	Case 3	Case 4	Case 5
General information						
Country	China	Togolese	Korea	Japan	Japan	Brazil
Gender	Female	Male	Male	Male	Female	Female
Gestational age	28w	Postmature	Full-term	38w	Full-term	Full-term
Age till now	21y	15y	4y	10y	19y(died of cardiac failure)	6y 6 m
Weight(kg)	12.0	12y 8 m: 18.2	11.5	10.4	2y: 6.8	2y 11 m: 8.8
Height(cm)	85.0	12y 8 m: 124.0	88.0	93.8	2y: 74.5	2y 11 m: 83.0
Initial abnormality						
Onset of abnormality	1 m	3w	1y	2 m	1 m	6 m
Initial manifestation	Sclerotic skin	Sclerotic skin; abdominal pigmentation	Growth retardation; hair loss; abdominal skin color changes	Hard skin; failure to thrive	Skin sclerosis; joint contracture	Progressive facial changes; thinning hair; failure to thrive
Cardiovascular syetem	Left atrial and left ventricular enlarge-ment; left ventricu-lar contraction and diastolic dysfuncti-on; moderate mitr-al regurgitation; ca-lcification in the aortic valve and bilateral coronar-y arteries	Left ventricular enlargement	Calcification of aortic and mitral valves	Rapid pulse rate; high blood pressure; bilateral obstruction of the supraclinoid portions of the internal carotid arteries	Not found	Tricuspid reflux
Brain and cerebrovascular	Encephalatrophy	Atrophy cerebral- cortical bi frontal	Not found	Small infarctions in the bilateral frontal Areas; a large infarction in the right parietal area	Old infarctions in both cerebral hemispheres	Not found

HGPS is characterized by accelerated ageing occurring up to seven times faster than normal [6], with a median life expectancy of only 14.6 years[3]. Our patient at 21 years of age is the oldest reported case of HGPS associated with typical mutations. In our opinion, appropriate cardiovascular intervention can improve survival in our patient. Based on our preliminary understanding of the disease, we speculated that progerin aggregation in patients with HGPS accelerates foetal growth and development, which result in premature birth, as observed in our patient. However, among the 23 cases of HGPS described in the current literature, we observed that patients with a history of preterm birth accounted for a low percentage (approximately 17.4%) of all cases, which was inconsistent with the presumed effect of progerin on the foetus (Table 1, Supplementary Table 1). We subsequently explored more data and observed that studies have confirmed that progerin did not affect in utero foetal growth and development, which suggests that preterm birth is an isolated phenomenon and does not characterise HGPS. Most of the 23 patients described in the literature developed initial symptoms within the first year of life and typically presented with skin changes, such as scleroderma. Our patient also had scleroderma-like skin changes one month after birth.

Rapid ageing of the cardiovascular system is a key feature of HGPS. Most case reports do not describe the progression of cardiovascular disease in patients with HGPS. We compared the clinical presentation of our patient between her two hospital admissions and have discussed this complication in detail. Evaluation at the time of the patient’s first admission revealed only reduced left ventricular diastolic function, mild mitral regurgitation, and aortic arch and coronary artery calcification; notably, the ventricular diameter and left ventricular systolic function were normal (left ventricular diameter 33 mm, EF:55%). However, the patient developed brain atrophy and significant ischaemic cardiomyopathy within a short span of the next 5 years, with left atrial and ventricular enlargement, left ventricular systolic dysfunction in addition to previous diastolic dysfunction, aggravation of the degree of mitral regurgitation, and diffuse vascular calcification involving multiple arterial beds. Rapid deterioration of cardiovascular function emphasises the importance of early diagnosis and prompt intervention to avoid serious cardiovascular complications associated with HGPS.

HGPS can lead to accelerated atherosclerosis with consequent fatal heart attacks or severe stroke [7]. Our patient also had a history of a heart attack. During the initial admission, the patient had a history of upper respiratory tract infection before she presented with chest pain. Although distinctive craniofacial features were observed on evaluation upon admission, owing to limited understanding of HGPS, we did not consider the possibility of coronary heart disease in this case, and based on the patient’s age, history, symptoms, and findings of elevated white blood cells and serum troponin I levels, we suspected viral myocarditis. During the second admission, in conjunction with the typical HGPS phenotype and echocardiographic findings, NSTEMI was retrospectively considered the likely cause of the patient’s chest pain during the first admission; however, the patient’s family refused coronary angiography to confirm this impression. Therefore, we emphasise that clinicians should consider coronary heart disease in the differential diagnosis of chest pain in patients with HGPS, regardless of age.

In conclusion, we report a case of HGPS (a rapidly progressive condition characterised by premature ageing of the cardiovascular system) associated with a typical C. 1824 C > T (P. Gly608Gly) mutation in the LMNA gene in a 21-year-old woman, who is reportedly the oldest patient with HGPS to have this mutation.

## Supplementary Information

Supplementary Material 1

## Data Availability

The data that support the findings of this study are available from Xiapu County Hospital but restrictions apply to the availability of these data, which were used under license for the current study, and so are not publicly available. Data are however available from the authors upon reasonable request and with permission of Xiapu County Hospital.

## References

[CR1] Pachajoa H, Claros-Hulbert A, García-Quintero X, Perafan L, Ramirez A, Zea-Vera AF. Hutchinson-Gilford Progeria syndrome: clinical and molecular characterization. Appl Clin Genet. 2020;13:159–64. 32943904 10.2147/TACG.S238715 PMC7481268

[CR2] Wang S, Yang Z, Xu Z, Chu Y, Liang Y, Wei L, Zhang B, Xu Z, Ma L. Clinical and genetic features of children with Hutchinson-Gilford progeria syndrome: a case series and a literature review. J Eur Acad Dermatol Venereol. 2021;35(6):387-387e391. 33590899 10.1111/jdv.17174

[CR3] Kreienkamp R, Gonzalo S. Metabolic dysfunction in Hutchinson-Gilford progeria syndrome. Cells. 2020;9(2):395. 32046343 10.3390/cells9020395 PMC7072593

[CR4] Ullrich NJ, Gordon LB. Hutchinson-Gilford progeria syndrome. Handb Clin Neurol. 2015;132:249–64. 26564085 10.1016/B978-0-444-62702-5.00018-4

[CR5] Rahman MM, Ferdous KS, Ahmed M, Islam MT, Khan MR, Perveen A, Ashraf GM, Uddin MS. Hutchinson-Gilford progeria syndrome: an overview of the molecular mechanism, pathophysiology and therapeutic approach. Curr Gene Ther. 2021;21(3):216–29. 33655857 10.2174/1566523221666210303100805

[CR6] Chu Y, Xu ZG, Xu Z, Ma L. Hutchinson-Gilford progeria syndrome caused by an LMNA mutation: a case report. Pediatr Dermatol. 2015;32(2):271–5. 25556323 10.1111/pde.12406

[CR7] Fatkin D. Left ventricular diastolic dysfunction in Hutchinson-Gilford progeria syndrome. JAMA Cardiol. 2018;3(4):334–5. 29466548 10.1001/jamacardio.2017.5377

