# Supplementary material for: Rare case of longevity in Hutchinson-Gilford progeria syndrome and literature review
Source: Orphanet J Rare Dis. 2025 Oct 7;20:506. doi: 10.1186/s13023-025-04022-6 (PMC12502240; doi:10.1186/s13023-025-04022-6)
Supplement: Supplementary file 1 — Supplementary Material 1 [file 13023_2025_4022_MOESM1_ESM.docx]

| **cases** |  | Case 1 | Case 2 | | Case 3 | Case 4 | Case 5 |
| --- | --- | --- | --- | --- | --- | --- | --- |
| **General information** | | | |  | | | |
| Country | China | Togolese | Korea | | Japan | Japan | Brazil |
| Gender | Female | Male | Male | | Male | Female | Female |
| Gestational age | 28w | Postmature | Full-term | | 38w | Full-term | Full-term |
| Age till now | 21y | 15y | 4y | | 10y | 19y(died of cardiac failure) | 6y 6m |
| Weight(kg) | 12.0 | 12y 8m：18.2 | 11.5 | | 10.4 | 2y: 6.8 | 2y 11m: 8.8 |
| Height(cm) | 85.0 | 12y 8m：124.0 | 88.0 | | 93.8 | 2y: 74.5 | 2y 11m: 83.0 |
| **Initial abnormality** |  |  |  | |  |  |  |
| Onset of abnormality | 1m | 3w | 1y | | 2m | 1m | 6m |
| Initial manifestation | Sclerotic skin | Sclerotic skin; abdominal pigmentation | Growth retardation; hair loss; abdominal skin color changes | | Hard skin; failure to thrive | Skin sclerosis; joint contracture | Progressive facial changes; thinning hair; failure to thrive |
| **Cardiovascular syetem** | Left atrial and left ventricular enlarge-ment; left ventricu-lar contraction and diastolic dysfuncti-on; moderate mitr-al regurgitation; ca-lcification in the aortic valve and bilateral coronar-y arteries | Left ventricular enlargement | Calcification of aortic and mitral valves | | Rapid pulse rate; high blood pressure; bilateral obstruction of the supraclinoid portions of the internal carotid  arteries | Not found | Tricuspid reflux |
| **Brain and cerebrovascular** | Encephalatrophy | Atrophy cerebral- cortical bi frontal | Not found | | Small infarctions in the bilateral frontal  Areas; a large infarction in the right parietal area | Old infarctions in both cerebral hemispheres | Not found |

| **cases** | Case 6 | Case 7 | Case 8 | | Case 9 | Case 10 | Case 11 |
| --- | --- | --- | --- | --- | --- | --- | --- |
| **General information** | | | |  | | | |
| Country | African | Belgium | China | | China | China | Brazil |
| Gender | Male | Male | Male | | Female | Male | Male |
| Gestational age |  | 37w | Full-term | | 39w | Full-term |  |
| Age till now | 12y | 2y | 9y | | 8.5y | 1y 11m | 11y |
| Weight(kg) | 12.8 | 7w: 3.1 | 14.0 | | 9.7 | 7.0 | 17.4 |
| Height(cm) | 107.0 | 7w: 48.0 | 102.0 | | 97.0 | 70.0 | 110.0 |
| **Initial abnormality** |  |  |  | |  |  |  |
| Onset of abnormality | 13m | 7w | 1m | | 2m | 46d |  |
| Initial manifestation | Hair loss; psychomotor regression | Failure to thrive; sclerotic skin | Skin appeared swollen and scleroder | | Sclerotic skin; subcutaneous fat atrophy | Sclerotic skin |  |
| **Cardiovascular syetem** | Not found | Not found | Not found | | Not found | Not found | Not found |
| **Brain and cerebrovascular** | Not found | Not found | Not found | | Not found | Not found | Not found |

| **Cases** | Case 12 | Case 13 | Case 14 | | Case15 | Case 16 | Case 17 |
| --- | --- | --- | --- | --- | --- | --- | --- |
| **General information** | | | |  | | | |
| Country | China | China | China | | China | China | China |
| Gender | Female | Female | Female | | Female | Male | Male |
| Gestational age | Full-term | 36w | 37w | | 34w | Full-term | Full-term |
| Age till now | 4y | 2y | 11m | | 6y | 15y | 3y |
| Weight(kg) | ＜3rd percentile | ＜3rd percentile | ＜3rd percentile | | ＜3rd percentile | ＜3rd percentile | ＜3rd percentile |
| Height(cm) | ＜3rd percentile | ＜3rd percentile | ＜3rd percentile | | ＜3rd percentile | ＜3rd percentile | ＜3rd percentile |
| **Initial abnormality** |  |  |  | |  |  |  |
| Onset of abnormality | 1m | 1m | 40d | | 2m | 1m | 1m |
| Initial manifestation | Sclerotic skin | Sclerotic skin | Sclerotic skin | | Sclerotic skin | hair loss | Sclerotic skin |
| **Cardiovascular syetem** | Not found | Not found | Not found | | Not found | Not found | Not found |
| **Brain and cerebrovascular** | Not found | Not found | Not found | | Not found | Cerebral infarction | Not found |

| **Cases** | Case 18 | Case19 | Case 20 | | Case 21 | Case 22 | Case 23 |
| --- | --- | --- | --- | --- | --- | --- | --- |
| **General information** | | | |  | | | |
| Country | Egyptian | China | China | | Colombia | Maroc | Brazil |
| Gender | Male | Female | Male | | Female | Female | Female |
| Gestational age | Full-term |  |  | |  |  |  |
| Age till now | 10y |  | 7m | | 14y | 5y | 30m |
| Weight(kg) | 11.5 |  |  | |  |  |  |
| Height(cm) | 102.0 |  |  | |  |  |  |
| **Initial abnormality** |  |  |  | |  |  |  |
| Onset of abnormality | 2y | At birth | 7m | | 2y |  |  |
| Initial manifestation |  | Shiny inelastic skin in the hip region | Sclerotic skin; failure to thrive | |  |  |  |
| **Cardiovascular syetem** | Not found | Not found | Not found | | Dilated cardiomyopathy; severe aortic and mitral insufficiency | Not found | Not found |
| **Brain and cerebrovascular** | Not found | Not found | Not found | | Not found | Not found | Not found |

**Supplementary Table 1** Literature review of Hutchinson-Gilford progeria syndrome.
